# Simultaneous Intrinsic and Extrinsic Parameter Identification of a Hand-Mounted Laser-Vision Sensor

**DOI:** 10.3390/s110908751

**Published:** 2011-09-09

**Authors:** Jong Kwang Lee, Kiho Kim, Yongseok Lee, Taikyeong Jeong

**Affiliations:** 1 Fuel Cycle System Engineering Technology Development Division, Korea Atomic Energy Research Institute, Daejeon, 305-353, Korea; E-Mails: leejk@kaeri.re.kr (J.K.L.); khkim5@kaeri.re.kr (K.K.); 2 Department of Electronic Engineering, Myongji University, Yongin-city, 449-728, Korea; E-Mail: leeys5867@mju.ac.kr (Y.L.)

**Keywords:** hand-mounted laser-vision sensor, parameter identification, particle swarm optimization

## Abstract

In this paper, we propose a simultaneous intrinsic and extrinsic parameter identification of a hand-mounted laser-vision sensor (HMLVS). A laser-vision sensor (LVS), consisting of a camera and a laser stripe projector, is used as a sensor component of the robotic measurement system, and it measures the range data with respect to the robot base frame using the robot forward kinematics and the optical triangulation principle. For the optimal estimation of the model parameters, we applied two optimization techniques: a nonlinear least square optimizer and a particle swarm optimizer. Best-fit parameters, including both the intrinsic and extrinsic parameters of the HMLVS, are simultaneously obtained based on the least-squares criterion. From the simulation and experimental results, it is shown that the parameter identification problem considered was characterized by a highly multimodal landscape; thus, the global optimization technique such as a particle swarm optimization can be a promising tool to identify the model parameters for a HMLVS, while the nonlinear least square optimizer often failed to find an optimal solution even when the initial candidate solutions were selected close to the true optimum. The proposed optimization method does not require good initial guesses of the system parameters to converge at a very stable solution and it could be applied to a kinematically dissimilar robot system without loss of generality.

## 1. Introduction

To measure the range data of objects under an unknown working environment, a range sensing device has been widely applied to various robotic applications [1–6]. In these researches, the range sensing device is usually installed on the robot hand and it is equipped with various sensors such as a camera(s), laser-vision sensor(s) and/or sonar(s). Since parameter identification, also referred to as calibration, is crucial to the system accuracy, it is considered as an important step before performing any measurement task. As for the robotic measurement system which integrates a robot with a range sensing device(s), generally, four different calibration procedures should be performed: sensor calibration, hand-to-sensor calibration, robot calibration, and base calibration [7]. In this work, we concentrated on the first two calibrations by assuming that we know the position of the calibration points exactly and that the geometric link parameter errors of the robot manipulator are negligible.

A laser-vision sensor (LVS) consisting of a CCD camera(s) and a laser stripe projector has been frequently used as an active ranging device [1–9] and a feature detection sensor [10]. It is mathematically modeled based on an optical triangulation principle [2] or a conversion matrix [3] which defines the geometrical relationship between the slit beam coordinates and their corresponding image coordinates. As for the model parameters of the LVS, there exist two parameter sets to be identified: the intrinsic parameters and the extrinsic parameters. As for a camera, the intrinsic parameters model the internal geometry and optical characteristics of the image sensor which determine how light is projected through the lens onto the image plane of the sensor. They consist of the focal length, the lens distortion coefficients, the optical center, and the magnification coefficient of the CCD cell [11]. As the extension of the intrinsic parameters of the camera, we considered the intrinsic parameters of the LVS which consist of the intrinsic camera parameters as well as laser stripe generator parameters such as a baseline distance and a projection angle with respect to a camera coordinate frame. The extrinsic parameters of the LVS are related to the position and orientation of the camera with respect to the robot hand coordinate system.

Most approaches to the calibration of a hand-mounted LVS have made use of the multi-stage technique [1–6], that is, the camera and laser parameter calibration stages were performed separately. The extrinsic parameters of the omnidirectional laser-vision sensor used in a free-ranging robot were identified after solving the intrinsic parameters based on the existing camera calibration method [3]. On the other hand, the extrinsic parameters were identified first to estimate the orientation and position of a camera with respect to a laser range finder and then the camera intrinsic calibration was performed [5]. The multi-stage technique, however, is known to have drawbacks such as error propagation.

Recently, the online re-calibration of a LVS which is mounted on a Cartesian carriage has been proposed to achieve good resolution and to avoid occlusions when the sensor geometry is modified online [8,9]. In the method, sensor parameters are determined by using the Bezier network without any calibration reference. In the robotic measurement system, the position and orientation of the sensor could be changed by manipulating a robot arm to avoid occlusion problems. Another approach is self-calibration, which aims to improve a practical implementation by introducing physical constraints such as a fixed point, a straight line, a circle, or a sphere to the system [1,4]. In these works, they used a commercial LVS, thus, the intrinsic parameters of the LVS were assumed to be known as *a priori*. However, it is analyzed in this paper that small amount of inexactness of model parameters could have a considerable effect on the measurement errors.

This paper is organized as follows. In Section 2, we address the hand-mounted laser-vision sensor model and analyze its measurement range and resolution. The parameter identification based on a particle swarm optimization is proposed in Section 3, and its performance is compared with conventional non-linear least square optimization in Section 4. Section 5 illustrates experimental results. The conclusion is given in the final section.

## 2. Hand-Mounted Laser-Vision Sensor Model

**Figure 1.**
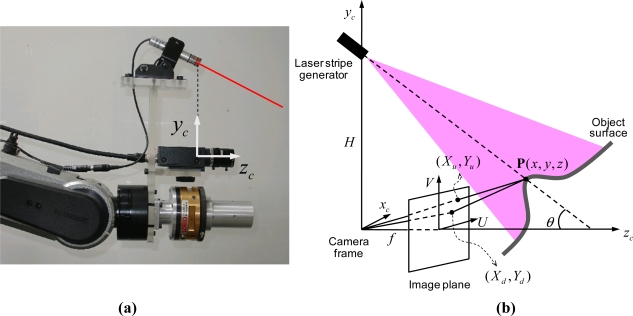
The laser-vision sensor model. The laser-vision sensor consists of a camera and a laser stripe generator. A 3D range data is obtained by using both the camera projection model and the optical triangulation principle. (**a**) Laser-vision sensor. (**b**) Laser-vision sensor geometry.

Figure 1 shows a schematic model for the hand-mounted laser-vision sensor (HMLVS). A 3D point **P**(*x*, *y*, *z*) in the camera coordinate system is transformed into an undistorted image coordinate (*X_u_*, *Y_u_*) by using a perspective projection with a pinhole camera geometry. Since the pinhole model is only an approximation of the real camera projection, a nonlinear lens distortion is considered to improve the measurement accuracy [2,11,12]. The distorted or true image coordinate (*X_d_*, *Y_d_*) is corrected by using the following equation:

(1) $$\begin{array}{c} X_{u}=f\frac{x}{z}=X_{d}/(1+\sum_{i=1}k_{i}r^{2i})\\ Y_{u}=f\frac{y}{z}=Y_{d}/(1+\sum_{i=1}k_{i}r^{2i}) \end{array}$$

where $r^{2}=X_{d}^{2}+Y_{d}^{2}$; *f* is the effective focal length of the camera; *k_i_* represents the coefficients of the radial lens distortion series. Since a sufficient accuracy can be achieved with a first-order distortion, we neglect the high order coefficients and use *k* = *k*_1_. The coordinate (*X_u_*, *Y_u_*) on the image plane is transformed to a 2D image pixel (*X_f_*, *Y_f_*) in a computer frame memory by using the magnification coefficients (*S_u_*, *S_v_*) and a center of the computer frame memory (*C_u_*, *C_v_*) as:

(2) $$\begin{array}{c} X_{f}=\frac{X_{u}-C_{u}}{S_{u}}\\ Y_{f}=\frac{Y_{u}-C_{v}}{S_{u}} \end{array}$$

Next, the 3D position of a point is computed through an optical triangulation principle. As shown in Figure 1, a laser stripe generator emits a plane of a beam with an angle *θ* relative to the *Z_C_* axis. The point **P**(*x*, *y*, *z*) on the object surface is projected onto the digitized image at the pixel (*X_f_*, *Y_f_*) and controlled by the effective focal length of the lens *f* and the baseline distance, *H*. Accordingly, the LVS can obtain 3D information in the camera coordinate system through measuring the image pixel coordinate (*X_f_*, *Y_f_*) which corresponds to the 3D coordinates **P**(*x*, *y*, *z*) of the illuminated laser point as:

(3) $${\;}^{c}\text{P}=\left[\begin{array}{c} x\\ y\\ z \end{array}\right]=\rho \left[\begin{array}{c} X_{f}\\ Y_{f}\\ f \end{array}\right]=\rho \text{U}$$

where $\rho =\frac{H}{Y_{f}+f\;\text{tan}\theta }$.

The intrinsic parameters of the LVS model include the intrinsic camera parameters, {*f*, *S_u_*, *S_v_*, *C_u_*, *C_v_*, *k*}, as well as the mounting parameters of the laser stripe generator with respect to the camera coordinate frame, {*H*, *θ*}. Because the LVS is installed on the last link of the robot manipulator, additional extrinsic parameters of the LVS, which define the position and the orientation of the camera frame with respect to the robot hand frame, should be considered.

A kinematic model of a robot manipulator can be modeled by using the Denavit-Hartenberg convention. Let ${}_{H}^{B}\text{T}$ be a 4 × 4 homogeneous transform matrix of a robot manipulator with n degree of freedom between the base frame and the hand frame, that is:

(4) $${}_{H}^{B}\text{T}={}_{1}^{0}\text{T}{}_{2}^{1}\text{T}\cdots {}_{n}^{n-1}\text{T}=\left[\begin{array}{cc} {\text{R}}_{N} & {\text{P}}_{N}\\ 0 & 1 \end{array}\right]$$

where ${}_{i+1}^{i}\text{T}$ is the homogeneous transformation matrix between two consecutive coordinate frames *i* and *i* + 1. If we denote the homogeneous transformation matrix between the hand frame and the camera frame as ${}_{C}^{H}\text{T}$:

(5) $${}_{C}^{H}\text{T}=\left[\begin{array}{cccc} r_{11} & r_{12} & r_{13} & t_{x}\\ r_{21} & r_{22} & r_{23} & t_{y}\\ r_{31} & r_{32} & r_{33} & t_{z}\\ 0 & 0 & 0 & 1 \end{array}\right]=\left[\begin{array}{cc} {\text{R}}_{C} & {\text{P}}_{C}\\ 0 & 1 \end{array}\right],$$

where *t_x_*, *t_y_* and *t_z_* denote the position of the camera frame relative to the hand frame; the elements, *r_ij_* in the rotation matrix **R**_C_ can be represented as a function of the Euler angle *R_x_*(*ω*), *R_y_*(*ϕ*), *R_z_*(*φ*) as:

(6) $${\text{R}}_{C}=\left[\begin{array}{ccc} \text{cos}\;\omega \;\text{cos}\;\phi \;\text{cos}\;\varphi -\text{sin}\;\omega \;\text{sin}\;\varphi & -\text{cos}\;\omega \;\text{cos}\;\phi \;\text{sin}\;\varphi -\text{sin}\;\omega \;\text{cos}\;\varphi & \text{cos}\;\omega \text{sin}\;\phi \\ \text{sin}\;\omega \;\text{cos}\;\phi \;\text{cos}\;\varphi +\text{cos}\;\omega \;\text{sin}\;\varphi & -\text{sin}\;\omega \;\text{cos}\;\phi \;\text{sin}\;\varphi +\text{cos}\;\omega \;\text{cos}\;\varphi & \text{sin}\;\omega \;\text{sin}\;\phi \\ -\text{sin}\;\phi \;\text{cos}\;\varphi & \text{sin}\;\phi \;\text{sin}\;\varphi & \text{cos}\;\phi \end{array}\right]$$

**Figure 2.**
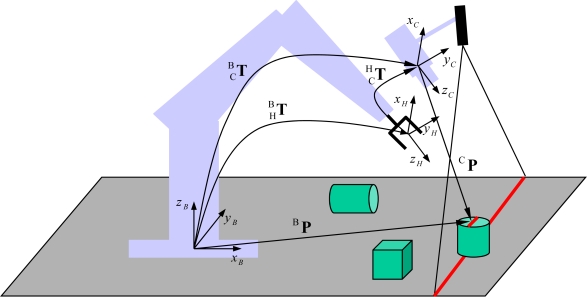
Kinematic model of the hand-mounted laser-vision sensor (HMLVS).

Since the transformation matrix between the robot base frame and the camera frame, ${}_{C}^{B}\text{T}$, can be represented as:

(7) $${}_{C}^{B}\text{T}={}_{H}^{B}\text{T}{}_{C}^{H}\text{T},$$

the position vector of the laser beam reflected by the object surface, ^B^**P**, is represented relative to the robot base coordinate frame by using Equations (3) and (7) as:

(8) $$\left[\begin{array}{c} {}^{B}\text{P}\\ 1 \end{array}\right]={}_{C}^{B}\text{T}\left[\begin{array}{c} {}^{C}\text{P}\\ 1 \end{array}\right]=\left[\begin{array}{cc} {\text{R}}_{N} & {\text{P}}_{N}\\ 0 & 1 \end{array}\right]\left[\begin{array}{cc} {\text{R}}_{C} & {\text{P}}_{C}\\ 0 & 1 \end{array}\right]\left[\begin{array}{c} \rho \text{U}\\ 1 \end{array}\right]$$

where ^C^**P** is the position vector of the reflected laser beam in the camera coordinate system. Therefore, the position of the reflected light with respect to the robot base frame (as shown in Figure 2) is calculated by using the following system model:

(9) $${}^{B}\text{P}=\rho {\text{R}}_{N}{\text{R}}_{C}\text{U}+{\text{R}}_{N}{\text{P}}_{C}+{\text{P}}_{N}.$$

**Figure 3.**
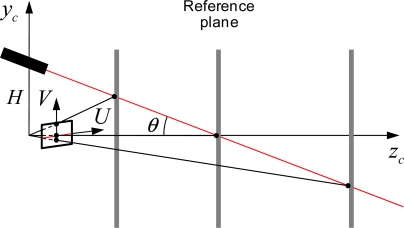
The geometric relation between a 3D illuminated laser point and its 2D image projection.

**Figure 4.**
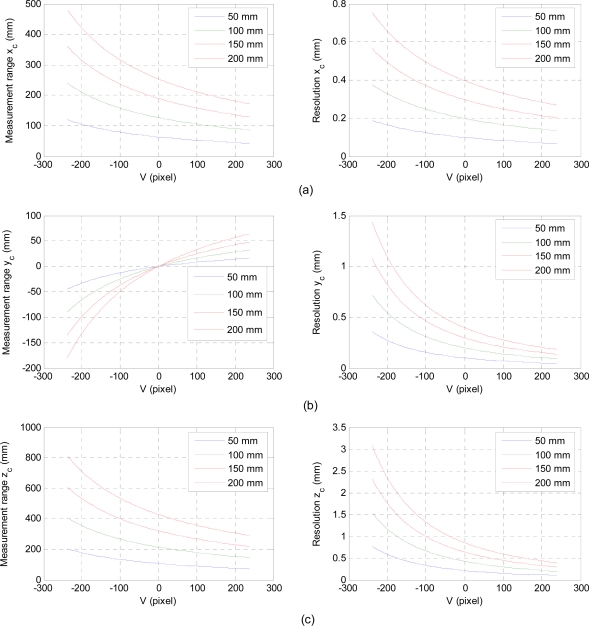
Measurement range and resolution for different baseline distances, where the projection angle is 25 degree.

**Figure 5.**
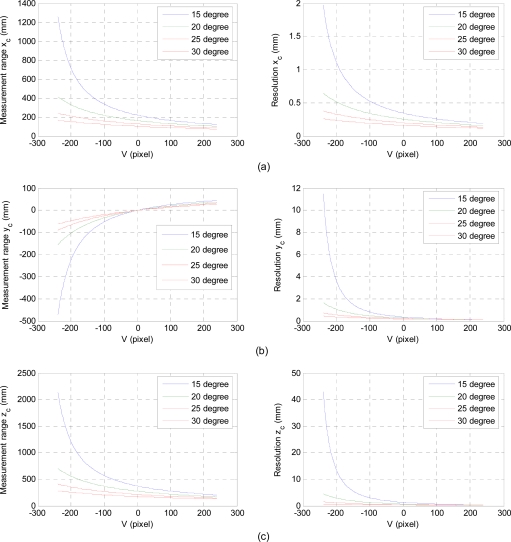
Measurement range and resolution for different projection angles, where the baseline H is 100 mm.

The baseline distance (*H*) and the projection angle (*θ*) affect the measurement range and resolution of the laser-vision sensor. Here, the resolution is defined as the displacement in the 3D real space per one pixel in the image plane. To investigate these characteristics, we consider a geometric relation as shown in Figure 3, where a reference plane moves parallel to an image plane along with *Z_c_* axis. A laser line illuminated horizontally on the reference plane shifts vertically as the distance between the image plane and the reference plane varies. According to Equation (3), the baseline distance acts as a scale factor for the 3D real coordinate of the illuminated laser line. Therefore, as *H* increases, the measurement range is increased by sacrificing the resolution as shown in Figure 4. For a horizontally illuminated laser line on the reference plane, the resolution about *x_c_* axis on the image coordinate system, Δ*x_c_* / Δ*X_f_*, is constant since Δ*Y_f_* / Δ*X_f_* is zero. As the reference plane approaches to the image plane, the illuminated laser line moves vertically upward direction about V axis on the image plane. In this case, the measurement range decreases while the sensor resolution increases as shown in Figures 4 and 5.

The baseline distance and the projection angle are design parameters of the laser-vision sensor, where they should be selected appropriately based on the requirements of target applications. If we design a sensor with a resolution of less than 1 mm/pixel, we could choose the baseline distance of 100 mm and the projection angle is 25 degree. In this case, measurement ranges about *x_c_*, *y_c_*, and *z_c_* are 155.0 mm, 122.3 mm, and 262.3 mm, respectively. To further increase the sensor resolution with the same configuration, measurement range should be decreased. This can be achieved by approaching the sensor to an object by manipulating a robot arm and its permissible distance can be observed by checking the laser line in the V axis of the image plane.

**Figure 6.**
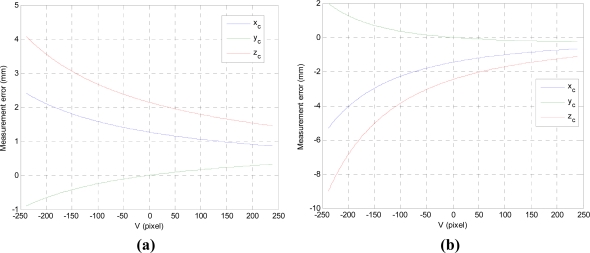
Measurement errors of the LVS with 1% inexact value of (**a**) the baseline and (**b**) the projection angle.

To investigate the effect of the inexactness of the baseline distance and the projection angle on the measurement errors, we carried out simulations. We assumed 1% inexactness of the baseline distance and the projection angle: the baseline distance is set as 100 mm for its real value of 99 mm, and the projection angle is 25 degree for its real value of 22.5 degree. Measurement errors are shown in Figure 6. It is important to note that these errors arisen from the inexactness are larger than the sensor resolution as shown in Figures 4 and 5. Moreover, since the baseline distance and the projection angle are measured with respect to the camera coordinate frame, they should be determined after the origin of the camera coordinates was obtained. In addition, mechanical errors arisen from manufacturing and assembling should be considered for better accuracy. So, they should be dealt with unknown parameters.

## 3. Parameter Identification

### 3.1. Objective Function

As a first step to identify the model parameters, we should define an objective function to be optimized. Let **q** be a vector consisting of the unknown intrinsic and extrinsic parameters of the HMLVS, that is:

(10) $$\text{q}={\left[f,S_{u},S_{v},C_{u},C_{v},k,H,\theta ,\omega ,\phi ,\varphi ,t_{x},t_{y},t_{z}\right]}^{T}$$

For a notational convenience, we rewrite **q** as:

(11) $$\text{q}={\left[q_{1},q_{2},\cdots ,q_{n}\right]}^{T}$$

where *n* is the number of unknown parameters (in this case, 14).

Searching boundary on parameters is set as:

(12) $$\forall q_{i}\in \left[q_{i}^{L},q_{i}^{U}\right]$$

where $q_{i}^{L}$ and $q_{i}^{U}$ denote the lower and upper bounds of *q_i_* respectively. Any reasonable interval which may cover the possible parameter values may be chosen as the bound of parameter *q_i_*.

In this work, we estimated a best-fit parameter vector q^*^ by minimizing the summed squared error of *m* nonlinear functions:

(13) $${\text{q}}^{*}=\underset{\text{q}}{\text{arg min}}F(\text{q})=\frac{1}{2}\sum_{i=1}^{m}{\left(f_{i}(\text{q})\right)}^{2}$$

where *f_i_* (**q**) ||*E_i_*||_2_ is a Euclidean norm of the error vector **E** which is given by:

(14) $$E=\text{P}-\rho {\text{R}}_{N}{\text{R}}_{C}\text{U}-{\text{R}}_{N}{\text{P}}_{C}-{\text{P}}_{N}.$$

### 3.2. Optimization Techniques

We applied two optimization techniques: nonlinear least squares optimization (NLSO) and particle swarm optimization (PSO). In the following paragraphs, we introduce them in brief. NLSO is a popular algorithm that is frequently used to find the minimum of a multivariate function represented as the sum of squares of the nonlinear functions. Among the various NLSO algorithms, we used the Levenberg-Marquardt algorithm (LMA) which is also referred to as the damped Gauss-Newton Method [13]. The LMA starts with an initial candidate solution. Given a current solution vector **q***_k_*, the LMA generates the next solution vector **q***_k_* _+ 1_ by using the following equation:

(15) $${\text{q}}_{k+1}={\text{q}}_{k}+\Delta {\text{q}}_{k}$$

where a vector of adjustments for the unknowns, Δ**q***_k_*, is computed as:

(16) $$\Delta {\text{q}}_{k}=-{\left[\nabla^{2}f({\text{q}}_{k})\right]}^{-1}\nabla f({\text{q}}_{k})$$

This process is repeated until *F* (**q***_k_*) or Δ**q***_k_* is sufficiently small; a maximum number of iterations are completed. In the LMA, the Hessian matrix is approximated as:

(17) $$\nabla^{2}f({\text{q}}_{k})={\text{J}}_{k}^{T}{\text{J}}_{k}+\lambda_{k}I$$

and the gradient is computed as:

(18) $$\nabla_{f}({\text{q}}_{k})={\text{J}}_{k}^{T}f({\text{q}}_{k})$$

where **J***_k_* is a Jacobian matrix which contains the first derivatives of the error vectors. The damping parameter *λ_k_* is a positive coefficient and it has several effects. When the current solution is far from the correct one, a large damping parameter is chosen so that the procedure tends toward the slow-convergent steepest descent method. On the other hand, when the current solution is close to the correct one, the damping parameter decreases and the LMA behaves like a Newton method. In this work, we used a public domain MINPACK [14] after slightly modifying the package to calculate the Jacobian matrix by using a forward-difference approximation.

The second identification technique considered is a particle swarm optimization (PSO) [15,16]. The PSO is a population-based evolutionary algorithm which is inspired by the social behavior of birds flocking for food. The position of a bird, also referred to as a particle, represents the current solution to the optimization problem. The PSO utilizes swarm intelligence to find the best place in the search. During each epoch, all the particles are accelerated toward their own best position and the global best position found so far by the swarm. This is achieved by calculating a new velocity of each particle ( $v_{i}^{t+1}$) according to three observations: its current velocity ( $v_{i}^{t}$), the distance between each particle’s current position ( $q_{i}^{t}$) and its previous best position ( $p_{i}^{t}$), and the distance from the global best position ( $p_{g}^{t}$) in the swarm:

(19) $$v_{i}^{t+1}=\omega v_{i}^{t}+c_{1}r_{1}(p_{i}^{t}-q_{i}^{t})+c_{2}r_{2}(p_{g}^{t}-q_{i}^{t})$$

where *i* is a particle index; *ω* is an inertia weight; *c*_1_ and *c*_2_ represent the weighting factors that pull each particle toward its previous best position and the global best position; *r*_1_ and *r*_2_ are random numbers uniformly distributed on the interval [0,1].

The inertia weight plays an important role to balance the global and local search abilities; a large inertia weight facilitates a global exploration, while a small one tends to facilitate a local exploration. A preferred weighting function, where the inertial weight is linearly decreased as the iteration proceeds, is described as:

(20) $$\omega =\omega_{\text{max}}-\frac{\omega_{\text{max}}-\omega_{\text{min}}}{T}t$$

where *ω*_max_ is the initial weight; *ω*_min_ is the final weight; *T* is the maximum iteration number; *t* is the current iteration number.

Once the new velocity of each particle is determined by using Equation (19), the particles update their position using the following equation:

(21) $$q_{i}^{t+1}=q_{i}^{t}+v_{i}^{t+1}$$

**Figure 7.**
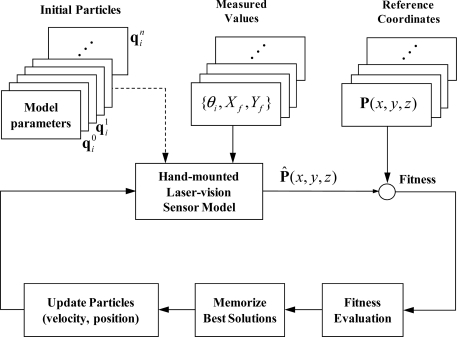
Schematic diagram of the PSO-based parameter identification.

In this way, the algorithm could converge toward a global solution of the given problem. The evolution is continued until the fitness value reaches the preset value or the maximum iterations are reached. Figure 7 shows how the PSO-based parameter identification works and the evolutionary optimization steps of the PSO are given below:

Step 1: Generate swarm and initialize particles in the swarm with random positions and velocities.
Step 2: For each particle, evaluate the fitness function.
Step 3: Memorize best solutions and a global best solution in the swarm.
Step 4: For each particle, update position and velocity by using Equations (19) and (21).
Step 5: Repeat Step 2 until predefined conditions are satisfied.

## 4. Simulations

In this section, we perform simulations to compare the performance of the two optimization techniques. Since the exact solution is known in the case of a simulation experiment, it is possible for us to compare the algorithms as to how the found solutions are close to the true optimum. At first, the synthetic data was generated as follows: given pre-specified laser-vision sensor model parameters and a certain robot pose, we compute 100 sets of data using Equation (9) in which 3D coordinates of the laser points with respect to the robot base frame correspond to randomly generated 2D image coordinates in the pixel frame. The first 50 sets of data are used to identify the model parameters while the other 50 sets of data are used to evaluate the fitness of the found solutions.

In order to investigate the influence of the initial candidate solutions on the convergence performance, we used a control parameter, *s* which determines the size of the initial parameter bounds. Accordingly, the initial solutions are randomly generated from within a certain parameter bound as:

(22) $$q_{i}=q_{i0}\times [1+(\text{rand}()-0.5)\times s]$$

where the function, rand(), calculates a uniformly distributed random number in the range [0,1]; *q_i_*_0_ is a *i*th nominal parameter used to generate the simulation data. Since some nominal model parameters such as *ω*, *ϕ*, *φ*, and *t_y_* are assumed to be zero, we select their bounds manually to avoid a null range.

Next, we set the control parameters of the two optimization techniques. In PSO, we used a swarm size of 50, a maximum inertia weight of 0.9, a minimum inertia weight of 0.4, a maximum velocity of 0.1, and a maximum iteration of 5,000. As for the LMA, the maximum iterations are set as 300 times the number of model parameters.

Since the applied optimization techniques set the initial guesses of the parameters in a random manner, they may seek out different minima depending on the initial conditions. Therefore, we performed several runs for each algorithm to evaluate the performance: 1,000 runs for NLSO and 10 runs for PSO. In addition to these algorithms, a random search (RS) was used only as a method to compare other algorithms. In the RS, an evaluation starts with parameters selected by using Equation (22) and when a better solution is found, it replaces the current solution. We performed 1,000,000 evaluations during a run.

The parameter identification results are listed in Table 1. As for the NLSO, it is possible to find sufficiently good solutions only when the initial estimates are close to the exact solutions. As the initial selection spaces increase, however, the technique has a considerably increased tendency to get trapped in the local minima or not to converge at all. Furthermore, even in the case with a small range of the initial parameters, the average root-mean-square error (rms) of NLSO is considerably high compared to that of the PSO. It indicates that the optimization problem considered has the characteristics of a highly nonconvex and multimodal landscape, even near the global optimum. On the contrary, the PSO consistently found a solution close to a true optimum, although the best fitness value slightly increased as the parameter bounds increased.

The average rms of the PSO with *s* = 0.2 (it means that the initial selection bounds are enlarged by up to ±20% of nominal parameters) is similar to that of the NLSO with *s* = 0.0001. This shows that the PSO can identify HMLVS parameters with small errors without the need for good initial estimates and that the enlarging spaces have only a marginal impact on the estimation accuracy.

**Figure 8.**
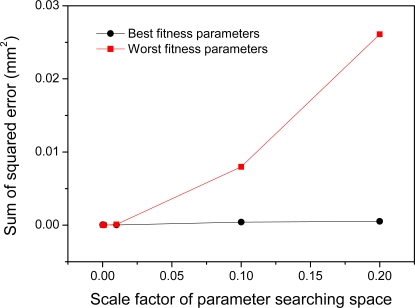
Fitness evaluation results of 50 sets of data not used in the parameter identification.

In order to validate and examine the reliability of the obtained model parameters, we calculate the sum of squared error of randomly generated 50 sets of data which is not used in the optimization by using the model parameters with both best fitness and worst fitness of the PSO. As shown in Figure 8, there is no significant difference between two results with different data sets even though the sum of squared error slightly increases as the parameter searching bounds enlarges. This shows the fact that the selection of input data does not affect on the reliability of the estimation accuracy.

## 5. Experimental Results

**Figure 9.**
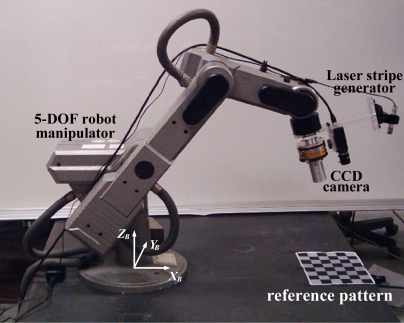
Hand-mounted laser-vision sensor and the planar calibration pattern.

Figure 9 illustrates the 5-DOF robot manipulator (SCORBOT ER-VII) and a reference object. A laser-vision sensor was installed at the last link of the robot manipulator. A laser reflection image is captured and digitized by a frame grabber (Meteor-II, Matrox) linked to a monochrome CCD camera (XC-55, Sony). A checker board pattern with a grid size of 30 mm × 30 mm is employed to provide reference positions. The left-top corner of the pattern is placed at the coordinate (700, 90, 0) mm with respect to the origin of the robot base frame. A robot controller controls the position and the velocity of the robot manipulator and it sends encoder readings of each joint to the PC through the RS-232C link.

As input data for the optimization techniques, we need three kinds of information: joint encoder readings, real coordinates of the reference points, and their corresponding image coordinates. The reference point is a corner point of the checker board pattern, which is given as priori information. The procedures for a data acquisition are as follows:

1. Adjust the robot so that a horizontal line of the laser stripe overlaps the corner points of the square pattern.
2. Capture an image and then extract the pixel coordinate of the reference point; record it in the memory of the PC.
3. Record all the joint angles of the manipulator sent from the joint controller.
4. Repeat (1)–(3) until we obtain the preset number of data sets.

In order to effectively extract a stripe of laser beam, we used a difference image, *D*(*x*, *y*) which subtracts one image frame without a laser beam, *F*′(*x*, *y*), from another image frame with a laser beam, *F* (*x*, *y*). This is achieved through toggling the on/off relay circuit used for providing power to the laser stripe projector:

(23) $$D(x,y)=\{\begin{array}{l} F(x,y)-F^{\prime }(x,y)\;\;\;\text{if}\;F(x,y)>F^{\prime }(x,y)\\ 0\;\text{if}\;F(x,y)>F^{\prime }(x,y) \end{array}$$

**Figure 10.**
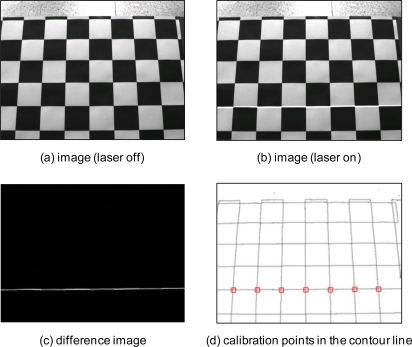
Detection result of the calibration points.

Calibration points are obtained through the matching process between a stripe of laser beam and cross-points of contour lines as shown in Figure 10(d). The developed algorithms including an image processing, robot control procedure, and two optimization techniques were implemented by means of the C++ language.

As described in the previous section, the considered parameter identification problem has highly nonconvex and multimodal characteristics such that the LMA often failed to find a good solution, even starting at an initial candidate solution which is near the true solution. Besides, the PSO consistently found a solution close to a true optimum regardless of the searching spaces. Therefore, we only considered the PSO for a parameter identification in the following experiments. As control parameters of the PSO, we used a swarm size of 50, a maximum inertia weight of 0.9, a minimum inertia weight 0.4, a maximum velocity of 0.1, and a maximum iteration of 5,000. The nominal values of the parameters are listed in Table 2 with their searching bounds, which are selected based on the specifications of the camera and the design parameters of the LVS. We determine the final parameters as those with the lowest fitness value from 20 different runs.

**Figure 11.**
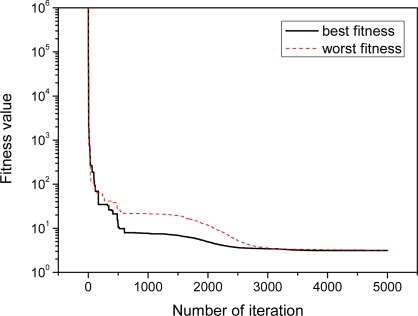
Convergence of the fitness value.

Figure 11 shows the convergence performance of the objective function, where the solid line shows the best fitness values from 20 different experiments, and the dotted line shows the worst fitness values.

Even though the initial particles (candidate solutions) are randomly generated from within the pre-defined range, these two curves are converged to a similar fitness value after approximately 3,000 iterations. This shows the fact that the PSO estimates the parameters with a small error. It takes about 30 seconds to execute the 5,000 generations.

**Figure 12.**
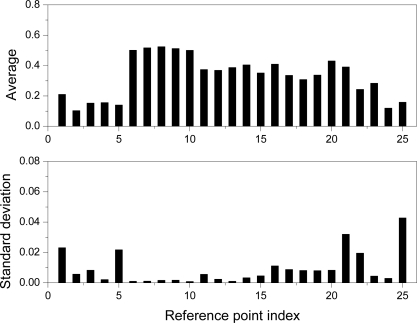
Calibration results.

**Figure 13.**
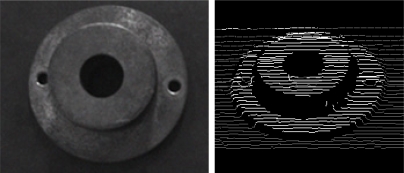
3D range measurement of a cylindrical object with holes.

Figure 12 shows the average values and standard deviation of the distance errors between the reference points and the calculated points of the 20 experimental results. The accuracy is computed based on a root-mean-square error (rms) and its average value of 20 experiments is 0.355 mm. By using the constructed HMLVS with parameters identified by the PSO, we measured the 3D range data of cylindrical object with holes, as shown in Figure 13.

To confirm the applicability of the proposed scheme, we carried out experiments measuring four corner points of the top surface of a gauge block whose size is 30 × 60 × 55 mm. A left-bottom of the block is placed at a point (0, −500, 0) mm with respect to the robot base frame. It is different position from the first experiment where the left-top corner of the pattern is placed at the coordinate (700, 90, 0) mm. We start to move the robot from the robot home position for each trial. We adjust the robot pose so as the laser stripe beam overlap the corner points of the top-side of the object. The measurement results are listed in Table 3, where *x*, *y*, *z* represent the Cartesian coordinate of the four corner points, and *x̄*, *ȳ*, *z̄* are the measured mean range data. *ē* and *σ* represent the mean value of the measurement error and standard deviation respectively. Even though it is different between the calibration region and the measurement region, maximum residual error with 10 trials was about 0.7 mm. This shows the suggested algorithm is robust against the measurement location.

## 6. Conclusions

In this paper we have proposed a new approach to the problem of a hand-mounted laser-vision sensor system calibration based on a particle swarm optimization. The laser-vision sensor, consisting of a camera with a nonlinear radial lens distortion and a laser stripe generator, was used as a sensor module of the robotic measurement system to measure the range data of an object in the robot base coordinate system; and it was modeled based on the forward kinematics and the optical triangulation principle. The intrinsic and extrinsic parameters of the hand-mounted laser-vision sensor were simultaneously identified through minimizing the overall residual errors between the known reference range data and the estimated data. Simulation and experimental results show that the considered parameter identification problem has highly nonconvex and multimodal characteristics, thus, the nonlinear least square optimizer often failed to find an optimal solution, even when the initial guesses of the model parameters were selected close to the true optimum. On the contrary, the proposed scheme based on the particle swarm optimizer consistently found a stable solution without any need for good initial guesses of the model parameters; thus, it could be a promising tool to identify the model parameters for a hand-mounted laser-vision sensor.

## Figures and Tables

**Table 1.** Estimation results with 50 sets of simulation data and with a different number of evaluations: 1,000,000 evaluations for RS, 1,000 runs for NLSO, and 10 runs for PSO.

**Optimization method**	**Root-mean-square error**	***s***				
		**0.0001**	**0.001**	**0.01**	**0.1**	**0.2**
RS	*E* (**q**)*_best_*	1.25E-2	7.48E-2	1.79	12.8	19.1
NLSO	*E* (**q**)*_best_*	2.01E-5	1.70E-4	7.50E-4	5.81E-3	2.49E-2
	*E* (**q**)*_worst_*	0.13	88.5	1.26E3	7.24E2	1.11E3
	*F* (**q**)*_mean_*	1.01E-1	6.75	4.67E2	6.20E2	6.00E2
	*F* (**q**)*_stdev_*	3.13E-2	20.5	48.6	4.43E2	4.60E2
PSO	*F* (**q**)*_best_*	1.69E-5	7.78E-5	3.79E-4	1.41E-3	2.55E-3
	*F* (**q**)*_worst_*	1.50E-4	2.72E-4	1.10E-3	1.04E-2	1.96E-2
	*F* (**q**)*_mean_*	9.34E-5	1.89E-4	8.65E-4	5.55E-3	1.16E-2
	*F* (**q**)*_stdev_*	9.11E-5	1.58E-4	6.05E-4	5.76E-3	1.13E-3

**Table 2.** Parameter identification results.

**Parameter**	**Nominal values**	**Parameter bounds of PSO**		**Best-fit parameters**
		**Lower**	**Upper**	
*f* (mm)	8	6	10	7.70
*S_u_* (mm)	0.0074	0.005	0.01	0.0074
*S_v_* (mm)	0.0074	0.005	0.01	0.0075
*C_x_*	320	300	340	323.8
*C_y_*	240	220	260	238.3
*k*	0	0	0.1	4.8E-4
*H* (mm)	140	120	150	137.3
*θ* (rad)	5*π*/36	0	*π*/4	0.47
*ω* (rad)	0	−*π*/16	*π*/16	−1.8E-3
*ϕ* (rad)	0	−*π*/16	*π*/16	−0.05
*φ* (rad)	0	−*π*/16	*π*/16	−6.8E-3
*t_x_* (mm)	60	40	80	63.2
*t_y_* (mm)	0	−10	10	−2.3
*t_z_* (mm)	−45	−60	−30	−46.2

**Table 3.** Measurement results for verification (mm).

**Ccorner points**	*x*	*y*	*z*	*x̄*	*ȳ*	*z̄*	*ē*	*σ*
left-top	60	−500	55	59.95	−499.56	55.34	0.58	0.15
right-top	60	−530	55	60.17	−530.01	54.65	0.39	0.14
right-bottom	0	−530	55	0.17	−529.48	55.14	0.61	0.21
left-bottom	0	−500	55	−0.51	−499.97	55.05	0.71	0.18
